# Characterization of Sv129 Mice as a Susceptible Model to *Leishmania amazonensis*

**DOI:** 10.3389/fmed.2019.00100

**Published:** 2019-05-29

**Authors:** Júlio Souza dos-Santos, Luan Firmino-Cruz, Tadeu Diniz Ramos, Alessandra Marcia da Fonseca-Martins, Diogo Oliveira-Maciel, Juliana Valente Rodrigues De-Medeiros, Suzana Passos Chaves, Daniel Claudio Oliveira Gomes, Herbert Leonel de Matos Guedes

**Affiliations:** ^1^Instituto de Biofísica Carlos Chagas Filho, Universidade Federal do Rio de Janeiro, Rio de Janeiro, Brazil; ^2^UFRJ Campus Macaé, Universidade Federal do Rio de Janeiro, Macaé, Brazil; ^3^Núcleo de Doenças Infecciosas/Núcleo de Biotecnologia- Universidade Federal do Espírito Santo, Vitória, Brazil; ^4^Núcleo Multidisciplinar de Pesquisa UFRJ – Xerém em Biologia, UFRJ Campus Duque de Caxias Professor Geraldo Cidade – Universidade Federal do Rio de Janeiro, Rio de Janeiro, Brazil

**Keywords:** leishmaniasis, *Leishmania amazonensis*, 129Sv, IL17-γδ T cells, susceptibility

## Abstract

Leishmaniasis is a complex of neglected diseases caused by parasites of the genus *Leishmania*, such as *Leishmania (Leishmania) amazonensis*, the ethiologic agent of diffuse cutaneous leishmaniasis in Brazil. In this work, we investigated a new experimental model of infection for *L. amazonensis*: the Sv129 mouse. First, we subcutaneously infected Sv129 mice with 2 × 10^5^ or 2 × 10^6^ *L. amazonensis* parasites of the Josefa strain. A progressive lesion developed for both inoculation doses, showing that Sv129 mice are susceptible, independent of parasite dose. We next investigated the mechanisms associated with the pathogenesis of infection. We did not observe an increase of frequency of interferon-gamma (IFN- γ)-producing CD4^+^ and CD8^+^ T cells, a phenotype similar to that seen in BALB/c mice. There was an increased of frequency and number of IL-17-producing γδ (gamma-delta) T cells in infected Sv129 mice compared to naïve SV129 and an increased frequency of this population compared to infected BALB/c mice. In addition, Sv129 mice presented high levels of both IgG1 and IgG2a, suggesting a mixed Th1 and Th2 response with a skew toward IgG1 production based on IgG1/IgG2a ratio. Susceptibility of the Sv129 mice was further confirmed with the use of another strain of *L. amazonensis*, LTB0016. In this work, we characterized the Sv129 mice as a new model of susceptibility to *Leishmania amazonensis* infection, during infection there was controlled IFN-γ production by CD4^+^ or CD8^+^ T cells and induced IL-17 production by γδ T cells.

## Introduction

Leishmaniasis is a complex of neglected diseases caused by protozoan parasites from the genus *Leishmania*, which is present in four continents (1). These diseases can be divided into cutaneous leishmaniasis (CL) or visceral leishmaniasis (VL), and VL can be fatal if not treated (2, 3). Mice are routinely used to model infections caused by the different species and strains of *Leishmania spp*. There are a few commonly used models which generally give polarized immune responses such as C57BL/6 mice and BALB/C mice (4, 5), however there are not yet any models that reflect the diversity of immune response as occurs in humans.

The Sv129 mouse strain was generated in Columbia University in 1928 and was commercially available from the Jackson's Laboratory in 1948. This strain has already been tested as a model for leishmaniasis caused by *L. amazonensis* (M2269 strain) (6). Using an inoculation dose of 1 × 10^6^ stationary-phase promastigotes, the authors described it as a resistant model, as these mice presented smaller lesions in the footpad compared to other experimental mouse strains. Lesions in the Sv129 mice also develop slowly, reaching just 1.2 mm after 8 weeks of infection (7, 8) with the *L. amazonensis* LTB0016 strain using an inoculation dose of 2 × 10^6^ stationary-phase promastigotes. As with the infections by *L. amazonensis*, Sv129 mice are also considered resistant against *L. major* infection (9), presenting a transitory lesion that resolves after 70 days of infection. However, further investigation in terms of the immune responses and additional timepoints used in *L. amazonensis* infection studies are required in order to conclude whether or not the Sv129 mouse strain is actually a resistant model.

Contradictory to what has been described by other authors, here, we would like to propose a new experimental model: Sv129 mice as a susceptible model of infection by *L. amazonensis*, the Josefa strain and also the LTB0016 strain. This new susceptible model could be useful in the modeling of the diverse human immune responses to infection and therefore, aid in the development of new drugs or vaccines against leishmaniasis.

## Materials and Methods

### Animals

Mice of the Sv129 strain were obtained from the Laboratory of Inflammation and Immunity at the Microbiology Institute of the Federal University of Rio de Janeiro (UFRJ). BALB/c mice were obtained from the Federal University Fluminense. All animals were kept in cages with commercial bedding and received filtered water and Nuvilab commercial feed.

All procedures performed with animals were previously approved by the Ethics Committee with the Use of Animals (CEUA) in Scientific Experimentation of the Health Sciences Center of UFRJ, under protocol number CEUA 157 and CCS082/18.

### Parasites

Two strains of *L. amazonensis* were used in this work: MHOM/BR/75/Josefa and MHOM/BR/77/LTB0016. Parasites were isolated in the amastigote form by puncture of lesions of infected BALB/c mice and maintained as promastigotes in M199 culture medium (Sigma-Aldrich) supplemented with 10% fetal bovine serum (FBS, Cultilab) and hemin (400 μg/200 ml) incubated at 26°C. To ensure infectivity, the parasites were only used in experiments up to the third passage of culture.

### Infection of Animals and Measurement of Lesions

Animals (five per group) were infected in the right hind footpad, using a syringe (HAMILTON) with 2 × 10^5^ or 2 × 10^6^ promastigotes of *L. amazonensis* Josefa and 2 × 10^6^ promastigotes of *L. amazonensis* LTB0016 in a volume of 20 μl. The footpad thickness was monitored using a *Mitutoyo* caliper. The thickness of the infected and uninfected footpads was measured prior to infection and lesion development was calculated as the difference between the thickness in vertical sense of the infected footpad and its thickness prior to infection. To calculate the area of lesion, vertical and horizontal thickness were measured and the values were multiplied.

### Determination of Parasite Loads by Limiting Dilution Assay (LDA)

Infected footpads were removed and placed in 70% alcohol for 1 min for disinfection. The spleens and draining lymph nodes were also removed. The footpads were weighed prior to maceration. These values were used to determine the parasitic load per gram mass. The organs were macerated in 1 mL of M199 medium containing 10% FBS; where flow cytometry was performed, the lymph nodes were macerated and washed with serum-free RPMI. A 96-well plate was pre-filled with 150 μl M199 medium supplemented with 10% FBS, and 50 μL of the cell suspension was placed in the first well, and a 1:4 serial dilution was performed by passing 50 μL of the dilution to the following well for a total of 24 dilutions for each sample. The plates were incubated in a bio-oxygen demand (BOD) incubator at 26°C for 7 to 14 days. After this time, the last well to show growth of promastigotes, as observed visually on a light microscope, was marked and used to calculate the total number of parasites present in the organ. The calculation used was as follows: Number of parasites = 4^x^/(mass of organs in grams); where *x* is the number of the last well in which parasites were observed.

### Flow Cytometry

Popliteal lymph nodes were removed and macerated as described earlier. Cells were quantified by light microscope using trypan blue and then plated at 1 × 10^6^ cells/well in a 96-well plate. Staining of intracellular and extracellular markers was performed following manufacturer's instructions. Briefly, cells were re-stimulated *ex vivo* for 4 h with phorbol 12-myristate 13-acetate (PMA; 20 ng/mL) plus ionomycin (1 μg/mL) in the presence of a Golgi complex inhibitor (brefeldinA) for intracellular cytokine analysis. Extracellular markers were stained, the cells were fixed and permeabilized to enable intracellular staining. Cells from lymph nodes of infected and control mice were phenotyped according to the criteria described by Cossarizza et al. (10). The antibodies used in this work were: anti-CD3-Pacific Blue (eBioscience) (1:200), anti-CD4-PeCy7 (eBioscience) (1:200), anti-B220-PercP 5.5 (eBiosciences) (1:200), anti-TCRγδ-FITC (eBioscience) (1:200) and anti-CD8-PercP (eBioscience) (1:200) for the extracellular markers, and anti-IL-17-APC (eBiosciences) (1:100) and anti-IFN-γ-APC (eBiosciences) (1:100) for the intracellular markers. Analysis was performed in the FlowJo software. Gate strategy for IFN-γ-producing cells on Supplementary Figure 7 and for IL-17- producing cells on Supplementary Figure 8.

### Determination of Antibody Concentrations

The titration was carried out by ELISA (Goat Anti-Mouse IgG1-UNLB:Cat. No. 1071-01, Goat Anti-Mouse IgG2a-UNLB: Cat. No.1101-01). Blood samples were collected, left at room temperature for 2 h, then centrifuged at 2,000 g to obtain serum. Total *Leishmania amazonensis* (LaAg) antigen at a concentration of 5 μg/mL diluted in PBS was used to coat the plate overnight. The next day, the antigen was discarded and the plate was blocked with Block Buffer (PBS with 5% heat-inactivated fetal calf serum (HIFCS, GIBCO Laboratories, Grand Island, NY, USA) and 0.05% Tween 20) for 1 h. The plate was washed three times with washing buffer (PBS with 0.05% Tween 20), the serum samples were diluted in Block Buffer and added to the plate for 1 h. The plate was washed five times with Washing Buffer and the secondary antibody specific for each isotype of interest was added for 1 h. The plate was washed again seven times with Washing Buffer and tetramethylbenzidine (TMB) was added. The reaction was stopped by the addition of HCl. The ratio of IgG1 and IgG2a isotypes was calculated by reducing OD from infected of OD from naïve and then dividing OD values for IgG1 by OD values for IgG2a.

### Statistical Analysis

The analysis of the results was done in the GraphPad Prism 5 program. Statistical tests of significance for differences between the groups of mice was determined by Two-way ANOVA or by Student's *T*-test. The values are represented as mean ± standard deviation of the mean.

## Results

### Sv129 Mice Develop Large Lesions and Have High Parasite Loads During *Leishmania amazonensis* Infection

**Figure 1:**
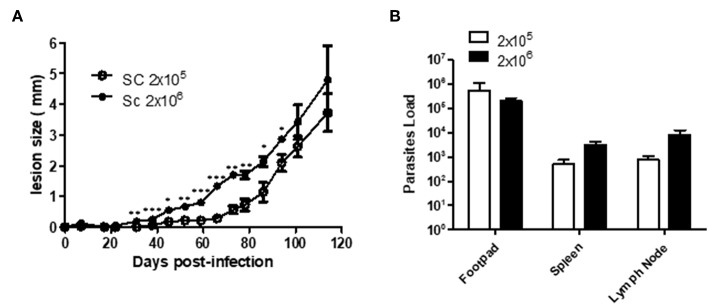
Sv129 mice develop large lesions with high parasite burdens in *Leishmania amazonensis* infection. Mice were infected with 2 × 10^5^ or 2 × 10^6^ *L. amazonensis* Josefa promastigotes in the right hind footpad. **(A)** The lesion development was monitored using a caliper on the indicated days until the 114th day post-infection. **(B)** The footpad, draining lymph node and spleen were removed on day 114th, macerated and used in a LDA to determine the parasite load/g of tissue. Data are representative of two independent experiments (mean ± standard deviation; *n* = 5). ***P < 0.01, **P < 0.01, *P < 0.05; assessed by Student's *T*-test.

**Figure 2:**
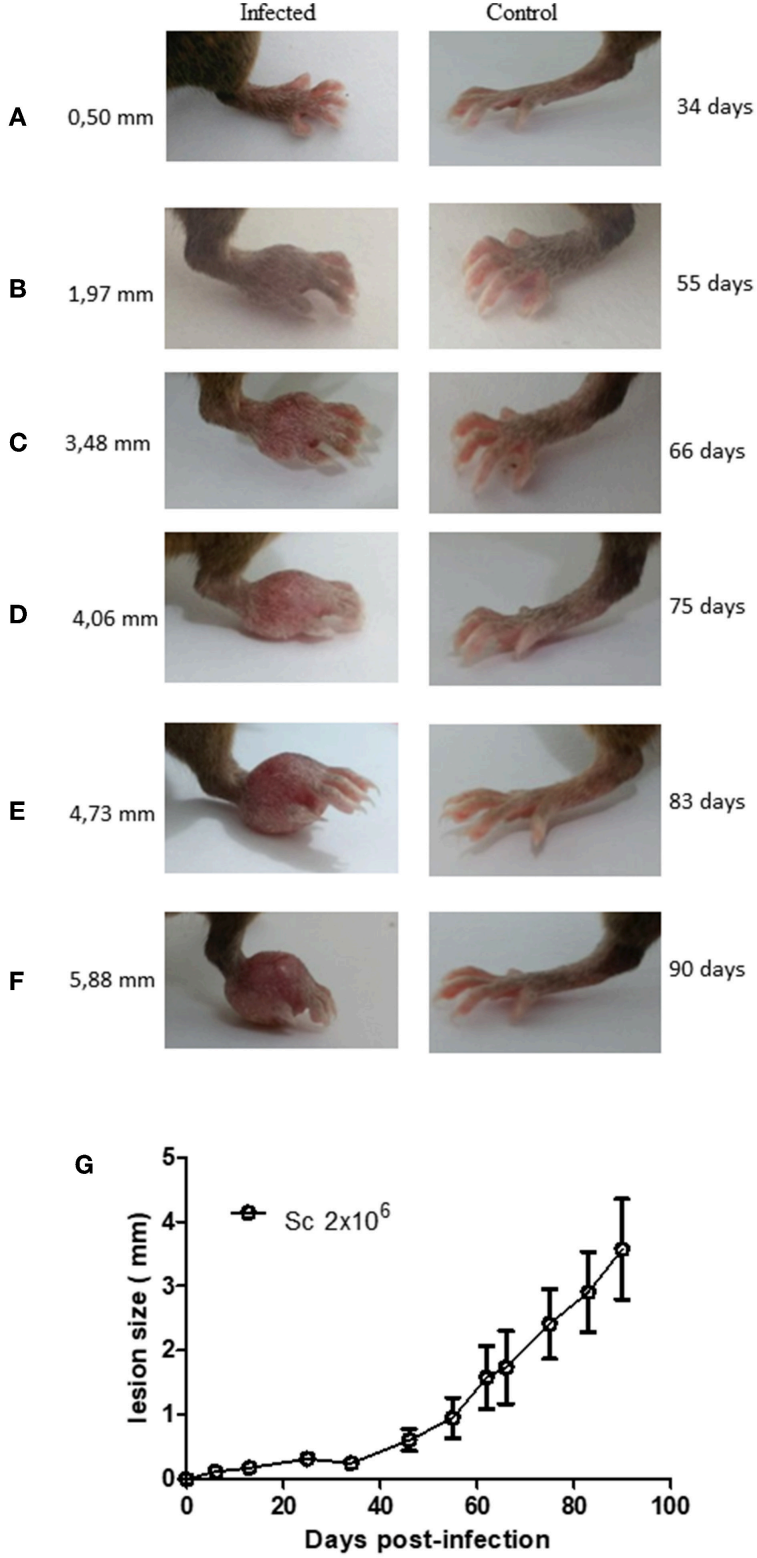
Lesion development in *Leishmania amazonensis*-infected Sv129 mice. Mice were infected with 2 × 10^6^ *L. amazonensis* Josefa promastigotes in the right hind footpad. Images were taken of the lesion development **(A–F)** and the footpad thickness was monitored using a caliper on the indicated days until the 90th day post-infection. **(G)** Data are representative of two independent experiments (mean ± standard deviation; *n* = 5).

A few studies have described Sv129 as a resistant mouse model for *L. amazonensis* infection using the M2269 strain of *L. amazonensis* (6), however, the time evaluated in these studies was too short to make this conclusion. In this study we evaluated the course of infection in Sv129 mice using the more virulent Josefa strain of *L. amazonensis*, and tested two inoculation doses of 2 × 10^5^ and 2 × 10^6^ promastigotes by the subcutaneous route. It was observed that the mice infected with the *L. amazonensis* Josefa strain presented progressive lesions with both inoculation doses (Figure 1A). However, there was a delay in the lesion development with a slow progression period of up to 31st days in mice infected with the 2 × 10^6^ inoculation dose and up to 45th days in those infected with 2 × 10^5^ promastigotes, after which the lesions developed more rapidly, reaching a peak of 4–5 mm at 114th days post-infection (Figure 1A). There was a difference in lesion development between the groups infected with different parasite doses from day 31st to 94th, but after day 100th there was no difference in the lesions between the groups. Despite the difference in the initial inoculation dose, the mice had no difference in the parasite load in the footpad, spleen and draining lymph node at day 114 post-infection (Figure 1B). We also evaluated area of lesion and similar profile were observed (Supplementary Figures 9, 10). Photographs of the lesion development with the 2 × 10^6^ inoculation were taken throughout the experiment (Figures 2A–F), as well as caliper measurements (Figure 2G), clearly showing the disease progression, indicating that Sv129 mice are a susceptible mice model for *L. amazonensis* infection. Photographs of the lesion development with 2 × 10^5^ inoculum displayed the same profile (Supplementary Figure 11).

### Evaluation of IFN-γ-producing CD4^+^ and CD8^+^ T Cells in Infected Sv129 Mice

**Figure 3:**
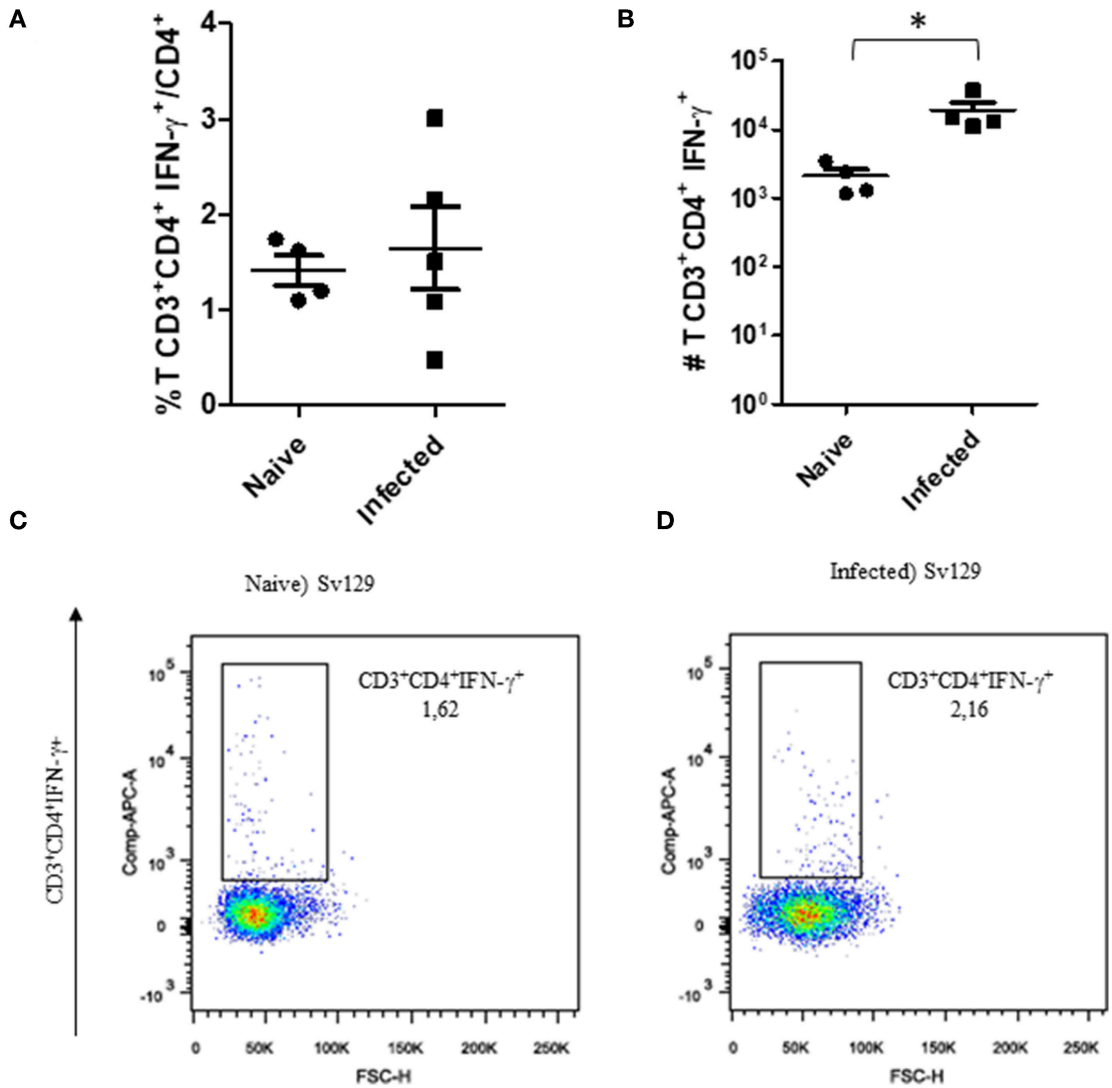
The IFN-γ-producing CD4+ T cell profile of infected Sv129 mice. Mice were infected with 2 × 10^6^ *L. amazonensis* Josefa promastigotes in the right hind footpad. Lymph node cells of infected Sv129 mice (90th day post-infection) and naive mice were plated at 1 × 10^6^ per well and re-stimulated for 4 h with PMA (20 ng/mL) plus ionomycin (1 μg/mL) and then stained for flow cytometry. The percentage of CD4^+^IFN-γ^+^ cells is presented in **(A)**, number of CD4^+^IFN-γ^+^ cells in **(B)** and the dotplot of the CD4^+^IFN-γ^+^ population of naive **(C)** and infected mice **(D)**. Data are representative of three independent experiments producing the same result profile (Mean ± standard deviation; *n* = 4–5). *P < 0.05; assessed by Student's *T*-test.

**Figure 4:**
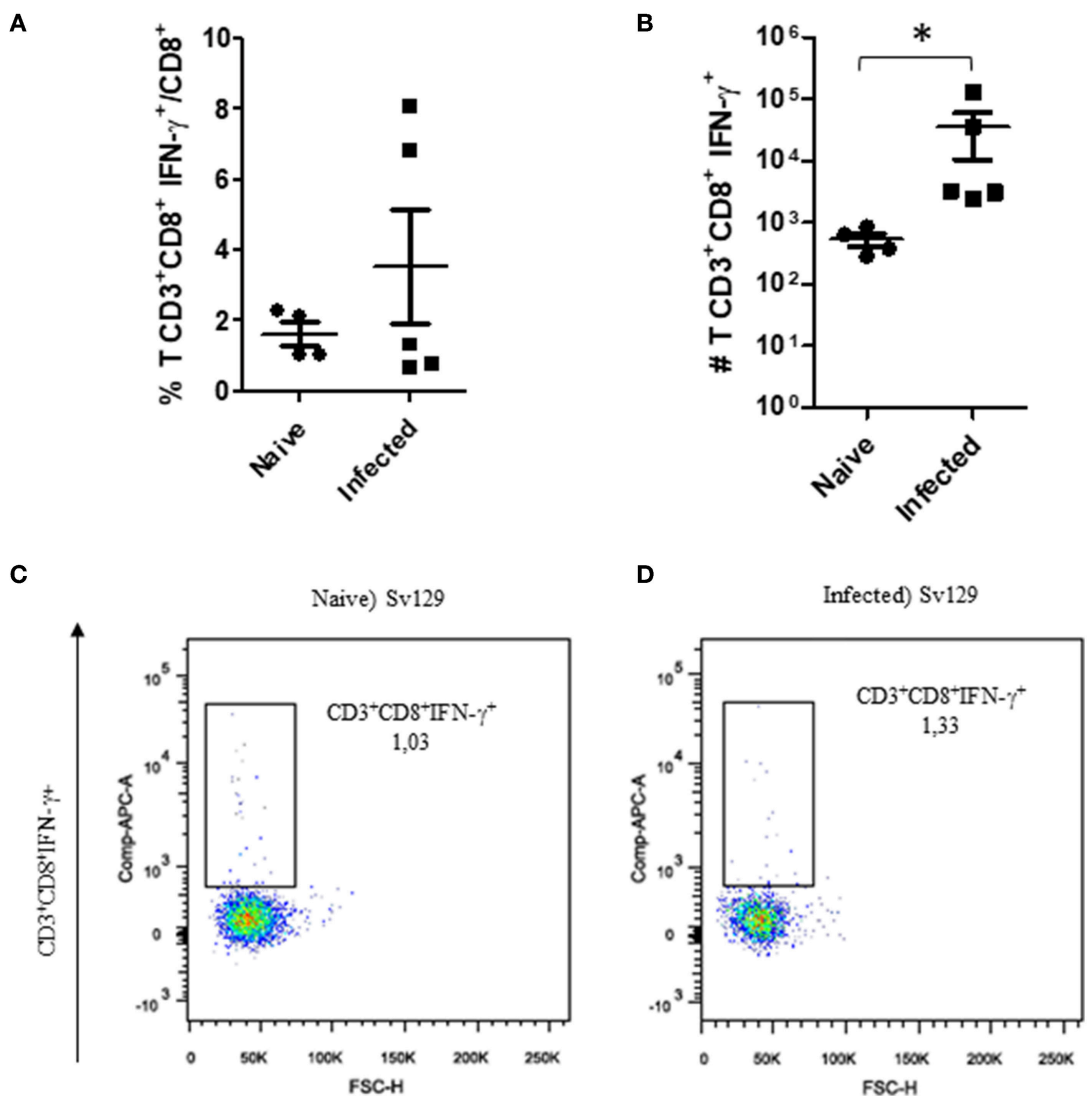
The IFN-γ-producing CD8+ T cell profile in infected Sv129 mice. Mice were infected with 2 × 10^6^ *L. amazonensis* Josefa promastigotes in the right hind footpad. Lymph node cells of infected mice (90th day post-infection) and naive mice were plated at 1 × 10^6^ per well and re-stimulated for 4 h with PMA (20 ng/mL) plus ionomycin (1 μg/mL) and then stained for flow cytometry. The percentage of CD8^+^IFN-γ^+^ cells is presented in **(A)**, number of CD8^+^IFN-γ^+^ cells in **(B)**, and the dotplot of the CD4^+^IFN-γ^+^ population of naive mice **(C)** and infected mice **(D)**. Data are representative of three independent experiments producing the same result profile (mean ± standard deviation, *n* = 4–5). *P < 0.05; assessed by Student's *T*-test.

The cellular response and production of IFN-γ has often been related to the control of *L. amazonensis* infection (4, 5, 11, 12). Therefore, to observe if the infection could prime the production of IFN-γ, we evaluated the production of IFN-γ by CD4^+^ and CD8^+^ T cells in infected and naive mice. We did not observe a change in the frequency of IFN-γ-producing CD4^+^ (Figure 3A, representative dotplots in Figure 3C for naive and Figure 3D for infected mice) and CD8^+^ T cells (Figure 4A, representative dotplots in Figure 4C for naive and Figure 4D for infected mice) in comparison to naïve mice suggesting that infection was not priming the cells to produce IFN-γ. The IFN-γ production observed was a backgroung production because is similar to naïve mice. When we evaluated the numbers of these cells, we observed a significant increase in the number of IFN-γ-producing CD4^+^ (Figure 3B) and CD8^+^ T cells (Figure 4B), this was due to the higher number of lymph node cells in infected mice (Supplementary Figure 1). The same phenotype of IFN-γ production was observed in BALB/c mice (Supplementary Figures 2, 3).

### Sv129 Mice Have an Increased Frequency and Number of IL-17-producing γδ T Cells in *Leishmania amazonensis* Infection

**Figure 5:**
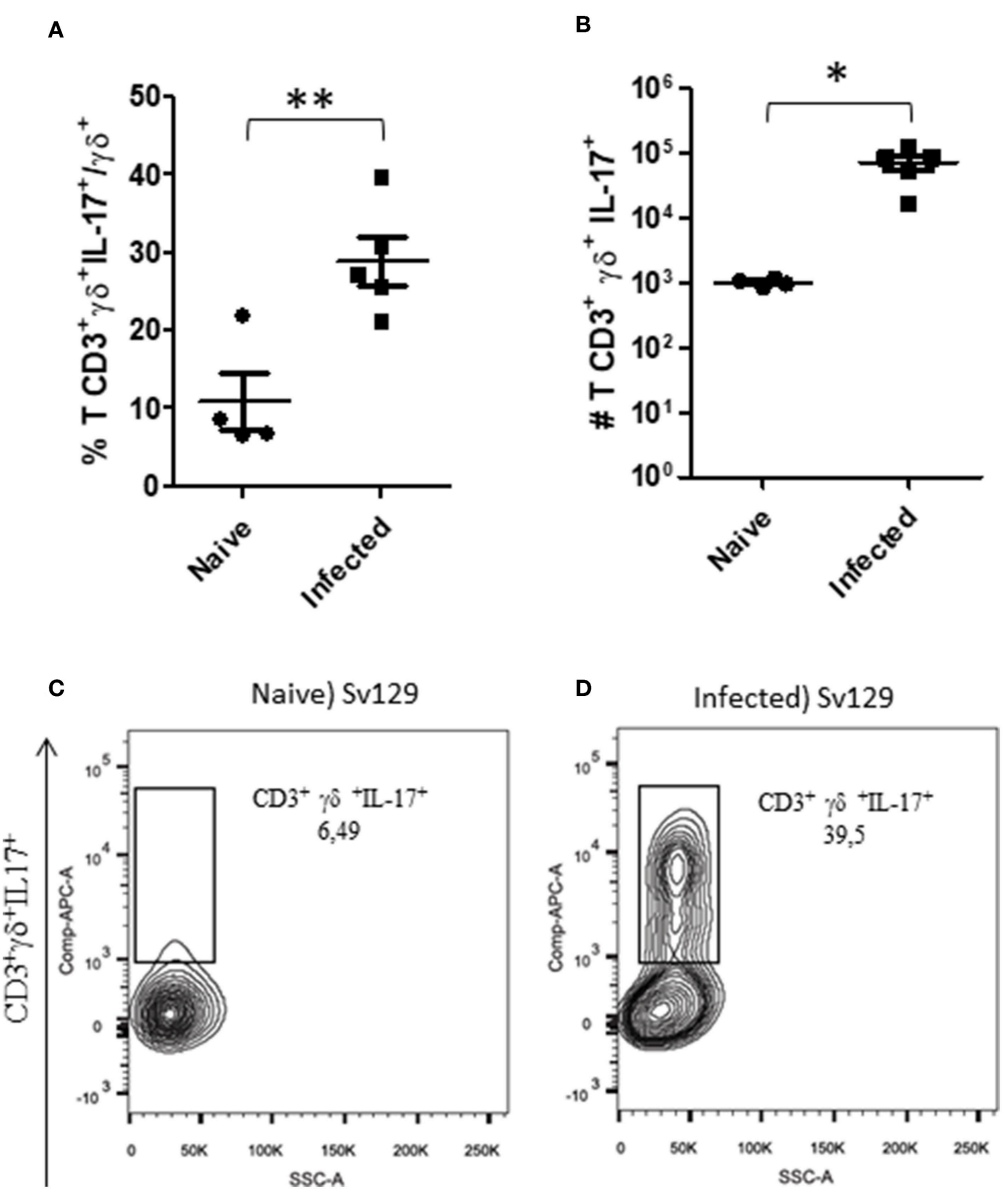
*L. amazonensis*-infected Sv129 mice present an increase in the frequency and number of IL-17-producing γδ T cells. Mice were infected with 2 × 10^6^ *L. amazonensis* Josefa promastigotes in the right hind footpad. Lymph node cells of infected mice (90th day post-infection) and naive mice were plated at 1 × 10^6^ per well and re-stimulated for 4 h with PMA (20 ng/mL) plus ionomycin (1 μg/mL) and then stained for flow cytometry. The IL-17-producing γδ T cells percentage is presented in **(A)**, number in **(B)** and the representative dotplot of the population of naive mice **(C)** and infected mice **(D)**. Data are representative of three independent experiments producing the same result profile (mean ± standard deviation; *n* = 4–5). **P < 0.001, *P < 0.01; assessed by Student's *T*-test.

Gamma-delta (γδ) T cells have been characterized as one of the major sources of IL-17 (13). IL-17 is one of the cytokines that has been described to be involved in susceptibility to cutaneous leishmaniasis (14, 15). We observed that the number of γδ T cells was significantly increased in both infected Sv129 and BALB/c mice (Supplementary Figure 4). We then investigated whether the capacity to produce IL-17 by these γδ T cells was altered in infected Sv129 mice. We observed that infected Sv129 mice had a higher frequency (Figure 5A, representative dotplots in Figure 5C for naive and Figure 5D for infected mice) and number (Figure 5B) of IL-17-producing γδ T cells than naive mice. We observed the same phenotype in BALB/c mice, with an increase in the frequency (Supplementary Figure 5A, representative dotplots in Supplementary Figure 5C for naive and Supplementary Figure 5D for infected mice) and number (Supplementary Figure 5B) of IL-17-producing γδ T cells. These results indicate that the Sv129 infection model is a susceptible model for *L. amazonensis* infection.

**Figure 6:**
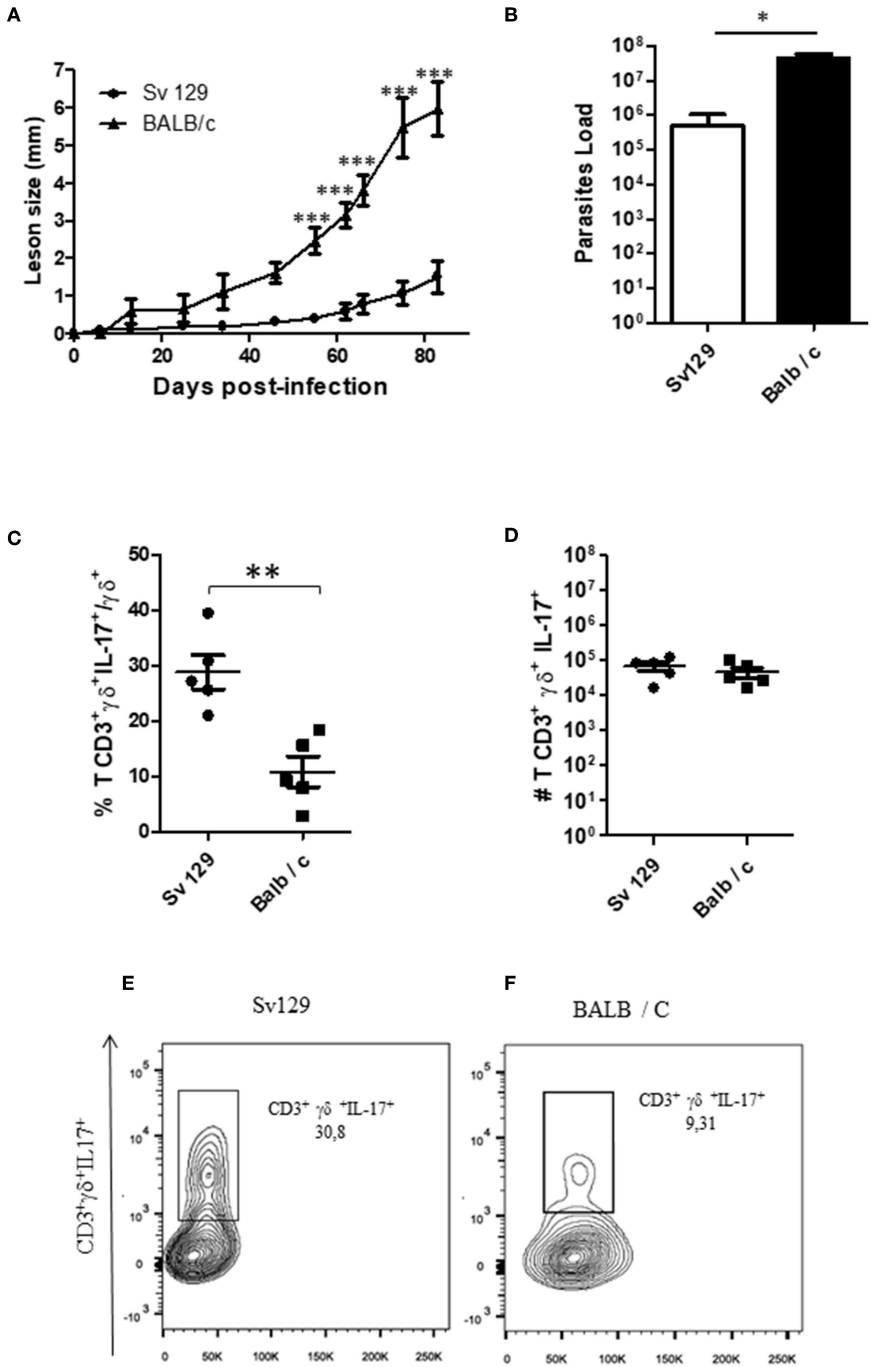
The lymph nodes of Sv129 mice have a higher frequency of IL17^+^ γδ T cells compared to BALB/c mice. Mice were infected with 2 × 10^6^ *L. amazonensis* Josefa promastigotes in the right hind footpad. The lesion development was monitored using a caliper on the indicated days until the 83th day post-infection. **(A)** Infected footpads were removed on day 83th, macerated and used in a LDA to determine the parasite load. **(B)** Lymph node cells of Sv129 and BALB/c mice were plated at 1 × 10^6^ per well and re-stimulated for 4 h with PMA (20 ng/mL) plus ionomycin (1 μg/mL) and then stained for flow cytometry. The IL-17-producing γδ T cell percentage is presented in **(C)**, number in **(D)** and the representative dotplots of the population of naive **(E)** and infected mice **(F)**. Data are representative of two independent experiments (mean ± standard deviation; *n* = 5). ***P < 0.001, **P < 0.01, *P < 0.05; assessed by Two-way ANOVA using Bonferroni's post-test. (disease score) and Student's *T*-test (bar graphs).

To confirm these data and compare the production of IL-17 by γδ T cells in both Sv129 and BALB/c mouse strains, we infected the mice at the same time with 2 × 10^6^ *L. amazonensis* (Josefa strain) promastigotes. Although the BALB/c exhibited faster lesion development (Figures 6A,B), we observed a greater frequency of IL-17^+^ γδ T cells in infected Sv129 mice (Figure 6C) indicating *in vivo* priming for IL-17 production in Sv129 mice in comparison to BALB/c. However, we did not observe differences in the number of IL-17-producing γδ T cells (Figure 6D), which is likely related to the higher number of lymph node cells in BALB/c in comparison to Sv129 (Supplementary Figure 6).

### Sv129 Induce a Mixed IgG1 and IgG2a Response During *Leishmania amazonensis* Infection

**Figure 7:**
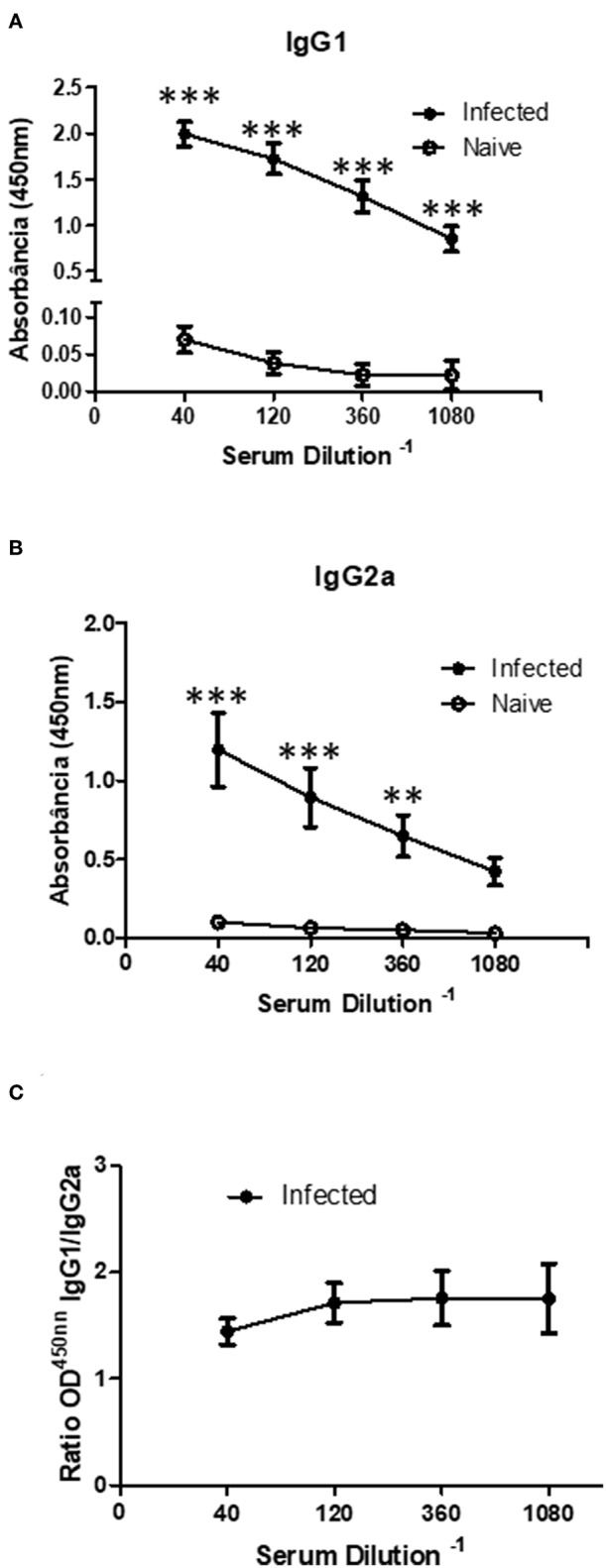
Infected Sv129 mice exhibit a mixed IgG1 and IgG2a serum antibody response. Mice were infected with 2 × 10^6^ *L. amazonensis* Josefa promastigotes in the right hind footpad. The sera of infected and non-infected mice (90th day post-infection) were taken and the antibody titration was assessed by ELISA. The total IgG1 **(A)** and IgG2a **(B)** specific for *Leishmania amazonensis* antigen was determined. The ratio of IgG1 and IgG2a isotypes was calculated **(C)**. Data are representative of two independent experiments (mean ± standard deviation; *n* = 3). ***P < 0.001; **P < 0.01 assessed by Two-way ANOVA using Bonferroni's post-test.

Since the Sv129 model had shown susceptiblity to infection in terms of the adaptive cellular response, we evaluated the specific antibody production, which our group has previously showed to be a pathogenic factor during *L. amazonensis* infection (16). We observed an increase in the concentration of both IgG1 (Figure 7A) and IgG2a (Figure 7B) revealing a mixed antibody response. The ratio of IgG1/IgG2a could be observed in Figure 7C, indicating a preference toward IgG1 production. This high antibody production could be related to pathogenesis of the infection in this model.

### Sv129 Mice Are Susceptible to Infection With Different Strains of *Leishmania amazonensis*

**Figure 8:**
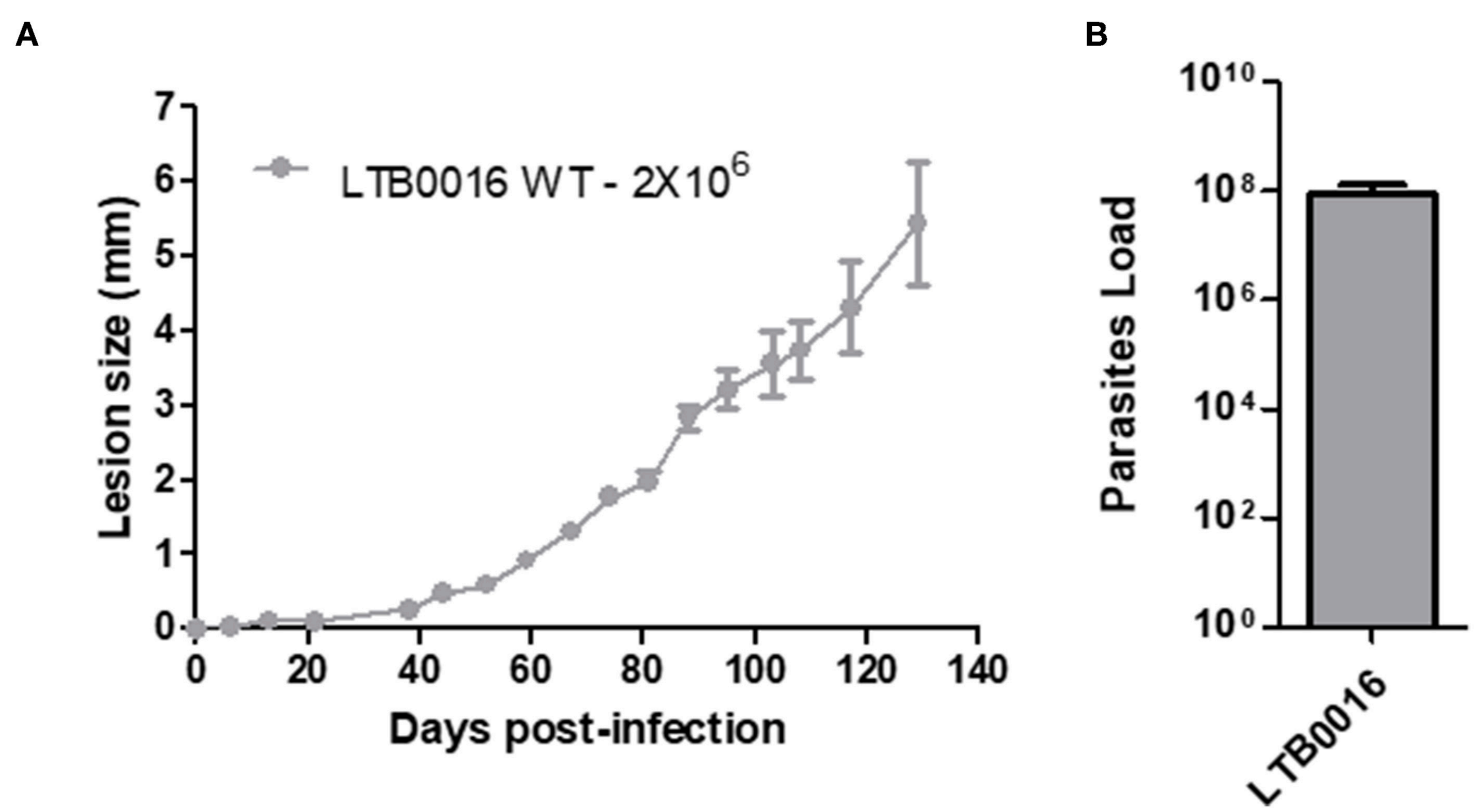
The susceptibility of Sv129 mice is not restricted to only one strain of *Leishmania amazonensis*. Mice were infected with 2 × 10^6^ promastigotes of a different strain of *Leishmania amazonensis*, LTB0016, in the right hind footpad. The lesion development **(A)** was monitored using a caliper on the indicated days until the 129th day post-infection. Infected footpads were macerated and used in a LDA to determine the parasite load. **(B)** Data are representative of two independent experiments (mean ± standard deviation; *n* = 5).

In order to further establish the Sv129 mice as a susceptible model of infection with *L. amazonensis*, the mice were infected with an inoculum of 2 × 10^6^ parasites of a different *L. amazonensis* strain (LTB0016), a strain which has been used before in Sv129 mouse infections (8). Mice were infected in the right hind footpad and the lesion development was evaluated weekly. This infection demonstrated the same profile as mice infected with the Josefa strain in terms of lesion progression (Figure 8A) and parasite loads in the footpad (Figure 8B). This result confirms that the susceptibility of Sv129 mice is not restricted to only one strain of *L. amazonensis*.

## Discussion

The leishmaniases are characterized as neglected diseases (1). The understanding of what immunological processes are involved in resistance and susceptibility to these diseases has been well-studied using experimental models, the main example of this is the Th1/Th2 response paradigm in *L. major* infection (4). In addition, experiments in the early phase of *L. major* infection has helped to understand the shaping of the immune responses that give rise to the resistant (C57BL/6) and susceptibility (BALB/c) models (5). However, human responses are not as polarized as animal models, and the responses can be dependent upon the immunological status and the infecting species or strain of *Leishmania spp*.

Here, we assess an established animal model for *Leishmania amazonensis*, the Sv129 mouse, which had previously been described as a resistant model. We investigated the immune responses during infection with the Josefa strain of *L. amazonensis*. We infected the mice subcutaneously with an inoculum of either 2 × 10^5^ or 2 × 10^6^ parasites, and observed that the Sv129 mice were susceptible to infection by this strain of *L. amazonensis* independent of the inoculation dose. The mice presented a progressive disease and no difference in the parasite load in the organs examined (Figures 1, 2). We sought to understand if the susceptibility of the Sv129 mice was restricted to only this strain of *L. amazonensis*. Therefore, we infected the Sv129 mice with the LTB0016 strain of *L. amazonensis* and monitored the lesion development over time. The Sv129 mice again developed a progressive lesion (Figure 8), which shows that the Sv129 mouse model can be used as a susceptible model for multiple strains of *L. amazonensis*.

The first description of the use of the Sv129 mouse model for *L. amazonensis* infection was by Serezani et al. (6), who made a comparison with the infection profile of BALB/c mice, thereby characterizing the Sv129 mice as partially resistant. The infection was carried out with initial inoculum of 1 × 10^6^ parasites. The second study used initial inoculum with 2 × 10^6^ parasites (8). These both studies used a dose of inoculum usually related to susceptibility to induce a Th2 phenotype on mice (17), however, the time of analysis of parasite loads and immune responses was very short (up to 2 months maximum) for investigating a chronic disease such as leishmaniasis. We suggest that in the beginning of infection there is a partial resistance phenotype, however, after 2 months of infection the disease becomes progressive. Based on that, the Sv129 mouse model in previous studies should only be considered as a partially resistant model.

Through the analysis of the cellular immune response of the Sv129 and BALB/c mice, although the infection increased the numbers of IFN-γ-producing cells, we could conclude that *in vivo* infection did not prime the proliferation of IFN-γ-producing CD4^+^ and CD8^+^ T cells sufficient enough to contain the infection (Figures 3, 4). Data from the literature have already shown that effective production of IFN-γ is required to controlleishmania parasites in experimental models of infection in C57BL/6 and BALB/c mice. Control of parasites is correlated with the production of IFN-γ by CD4^+^ T cells, whilst uncontrolled infections have an absence of IFN-γ in these mice (12, 18–20) using C57BL/6 mice have shown that CD4^+^ T cells can play a pathogenic role in *L. amazonensis* infection, in which mice with MHC-2 depletion and lymphocyte depletion, in transgenic mice lacking the recombinant activation gene 2 (RAG-2^−/−^ mice), were more resistant to infection, presenting smaller lesions in relation to wild-type animals. In addition, RAG-2^−/−^ animals reconstituted with CD4^+^ T cells of wild-type animals presented larger lesions, suggesting the pathogenic role of lymphocytes in this model. It is generally considered that, Th1 (12) and Treg (21) are associated to protection, and Th2 (4, 22, 23) and Th17 (24) are associated to pathology.

Interestingly, we observed that infected Sv129 mice failed to control the infection and exhibited large numbers of IL-17-producing γδ T cells compared to naive mice, which may be related to the susceptibility of these mice (Figure 5). A similar result was also observed in BALB/c mice (Supplementary Figure 5), however, the Sv129 mice are more competent to induce IL-17-producing γδ T cells in comparison to BALB/c (Figure 6). In *L major* infection, IL-17 knock-out mice in the BALB/c background have been shown to be resistant to infection (14). The source of IL-17 was not determined. In *L. mexicana* infection (15), showed a great and constant presence of Th17 cells throughout the experiment in the susceptible BALB/c mice. They also reported that lesions of *L. mexicana*-infected BALB/c and C57BL/6 mice showed a high expression of IL-17, which coincided with areas of CD4^+^ cells and NIMP-R14^+^ cells (neutrophils) within the chronic inflammatory infiltrate. These data suggested that IL-17, probably produced by CD4^+^ cells, induces inflammation and recruitment of neutrophils that are associated with chronic infection. Terrazas et al. (24) proposed that the IL-17A cytokine promotes susceptibility during experimental visceral leishmaniasis caused by *L. donovani*, by showing that IL-17A^−/−^ mice were highly resistant to infection, with decreased parasite loads in the liver and spleen. This result was associated with IFN-γ production by T cells and decreased accumulation of neutrophils and monocytes, resulting in a reduced number of granulomas. There was also a decrease in IL-4, IL-6, IL-10, and IL-13 in the late stages of infection. We demonstrate in our study that the γδ T cells are the source of IL-17 in this *L. amazonenis* infection.

Hypergammaglobulinemia is one of the hallmarks of leishmaniasis (25). Silva-Barrios et al. (26) showed through cell-specific ablation of endosomal TLR signaling in B cells that innate B cell activation by *L. donovani* is responsible for disease exacerbation through IL-10 and IFN type I production and for the promotion of hypergammaglobulinemia. Whilst Firmino-Cruz et al. (16) showed that B cells are related with lesion pathogenesis through the production of antibodies and IL-10 during *L. amazonensis* infection in BALB/c mice (27) showed that B cell-deficient JHD mice infected with *L. amazonensis* show a significantly reduced lesion compared to wild-type mice, suggesting that B cells promote exacerbation of the infection, and that antibodies may contribute to immunopathology. Here, we show that Sv129 mice present an increase of both IgG1 and IgG2a (Figure 7) indicating a mixed antibody response. This high antibody production could be related to pathogenesis of the infection in this model.

In the clinical presentations of leishmaniasis, the disease is associated with an increase of IL-17 (28–30), an increase of γδ T cells (31–35), and a production of antibodies (36). In this study, we have identified many immunological characteristics that are similar to that seen in the clinical presentations of the disease. Therefore, we suggest that the Sv129 mouse is an important model to study leishmaniasis further in terms of immunological responses and treatment options. We have also demonstrated for the firsttime that the increase of IL-17 is related to γδ T cells, which should be further investigated in human diseases.

## Conclusion

In this study, Sv129 mice were characterized as a susceptible model to *Leishmania amazonensis* through the failure to produce IFN-γ and the increase of antibodies and IL-17 production. These mice could enable additional studies to evaluate the role of IL-17 in *Leishmania* infection and could be an alternative to BALB/c mice as an experimental model to test drugs and vaccines.

## Ethics Statement

The Health Sciences Center Ethics Committee of Federal University of Rio de Janeiro (Comissão de Ética no Uso de Animais do Centro de Ciências da Saúde da Universidade Federal do Rio de Janeiro) approved the animal use under the protocol number IBCCF 157 and CCS082/18.

## Author Contributions

All authors listed have made a substantial, direct and intellectual contribution to the work, and approved it for publication.

### Conflict of Interest Statement

The authors declare that the research was conducted in the absence of any commercial or financial relationships that could be construed as a potential conflict of interest.

## Supplementary Material

Supplementary Figure 1 Lymph node cellularity, and frequency and number of CD4^+^ and CD8^+^ T cell populations from infected and naive Sv129 mice. Mice were infected with 2 × 10^6^ *L. amazonensis* Josefa promastigotes in the right hind footpad. Lymph node cells of infected mice and naive Sv129 mice were quantified using a Neubauer‘s chamber **(A)** (mean ± standard deviation; *n* = 4–5). Lymph node cells from infected mice and naive Sv129 mice were plated at 1 × 10^6^ per well and stained for flow cytometry; frequency and total of CD4^+^ cells **(B,C,F,G)** and frequency and total of CD8^+^ cells were stained **(D,E,H,I)**. Data are representative of four independent experiments (mean ± standard deviation; *n* = 4–5) ^*^*P* < 0.05; assessed by Student's *T*-test.

Supplementary Figure 2 Infected BALB/c mice have an increase in the number of IFN-γ-producing CD4^+^ T cells. Mice were infected with 2 × 10^6^ *L. amazonensis* Josefa promastigotes in the right hind footpad. Lymph node cells of infected and naive BALB/c mice were plated at 1 × 10^6^ per well and re-stimulated for 4 h with PMA (20 ng/mL) plus ionomycin (1 μg/mL) and then stained for flow cytometry. The percentage of CD4^+^IFN-γ^+^ **(A)** and number of CD4^+^IFN-γ^+^ **(B)** cells. Representative dotplots of the CD4^+^IFN-γ^+^ population **(C,D)** (mean ± standard deviation; *n* = 4–5). Data are representative of three independent experiments producing the same result profile. ^*^*P* < 0.05; assessed by Student's *T*-test.

Supplementary Figure 3 Infected BALB/c have an increase in the number of IFN-γ-producing CD8^+^ T cells. Mice were infected with 2 × 10^6^ *L. amazonensis* Josefa promastigotes in the right hind footpad. Lymph node cells of infected mice and naive mice were plated at 1 × 10^6^ per well and re-stimulated for 4 h with PMA (20 ng/mL) plus ionomycin (1 μg/mL) and then stained for flow cytometry. The percentage of CD8^+^IFN-γ^+^ **(A)** and number of CD8^+^IFN-γ^+^ **(B)** cells. Representative dotplots of the CD4^+^IFN-γ^+^ population **(C,D)**. Data are representative of three independent experiments producing the same result profile (mean ± standard deviation; *n* = 4–5). ^*^*P* < 0.05;assessed by Student's *T*-test.

Supplementary Figure 4 Infected Sv129 mice present an increase in the number of γδ T cells. Mice were infected with 2 × 10^6^ *L. amazonensis* Josefa promastigotes in the right hind footpad. Lymph node cells of infected mice and naive mice were plated at 1 × 10^6^ per well and re-stimulated for 4 h with PMA (20 ng/mL) plus ionomycin (1 μg/mL) and then stained for flow cytometry. The number of γδ T cells in infected and naive Sv129 mice **(A)** and BALB/c mice **(B)**. Representative of three independent experiments (mean ± standard deviation; *n* = 6–9). ^**^*P* < 0.001, ^*^*P* < 0.05; assessed by Student's *T*-test.

Supplementary Figure 5 Infected BALB/c mice show an increase in the frequency and number of IL-17-producing γδ T cells. Mice were infected with 2 × 10^6^ *L. amazonensis* Josefa promastigotes in the right hind footpad. Lymph node cells of infected mice and naive mice were plated at 1 × 10^6^ per well and re-stimulated for 4 h with PMA (20 ng/mL) plus ionomycin (1 μg/mL) and then stained for flow cytometry. The percentage **(A)** and number **(B)** of IL-17-producing γδ T cells. Representative dotplots **(C,D)**. Data are representative of three independent experiments producing the same result profile (mean ± standard deviation; *n* = 3). ^***^*P* < 0.0002, ^***^*P* < 0.0003 assessed by Student's T-test.

Supplementary Figure 6 Lymph node cellularity in Sv129 and BALB/c mice. Mice were infected with 2 × 10^6^ *L. amazonensis* Josefa promastigotes in the right hind footpad. Total lymph node cells of infected Sv129 and BALB/c mice were quantified using a Neubauer‘s chamber **(A)**. Data are representative of three independent experiments producing the same result profile (mean ± standard deviation; *n* = 5). ^*^*P* < 0.01; assessed by Student's *T*-test.

Supplementary Figure 7 Gating strategy used to analyze IFN-γ producing cells. Mice were infected with 2 × 10^6^ *L. amazonensis* Josefa promastigotes in the right hind footpad. Lymph node cells from infected and naive Sv129 mice were plated at 1 × 10^6^ per well and stained for flow cytometry to determine the percentage of IFN-γ-producing cells. Single cells were gated on CD3^+^ and CD4^+^ or CD8^+^, then on IFN-γ^+^.

Supplementary Figure 8 Gating strategy used to analyze IL17^+^ γδ T cells from the lymph node. Mice were infected with 2 × 10^6^ *L. amazonensis* Josefa promastigotes in the right hind footpad. Lymph node cells from infected and naive Sv129 mice were plated at 1 × 10^6^ per well and stained for flow cytometry to determine the percentage of IL17^+^ γδ T cells. Single cells were gated on CD3^+^ and γδ^+^, then on IL-17^+^.

Supplementary Figure 9 Evaluation of the lesion area of Sv129 mice in *Leishmania amazonensis* infection. Mice were infected with 2 × 10^6^ *L. amazonensis* Josefa promastigotes in the right hind footpad. **(A)** The lesion development was monitored horizontally using a caliper on the indicated days until the 114th day post-infection. **(B)** The lesion area was evaluated as described in Method. Data are representative of two independent experiments (mean ± standard deviation; *n* = 5). ^*^*P* < 0.05; assessed by Two-way ANOVA using Bonferroni's post-test.

Supplementary Figure 10 Evaluation of the lesion development of Sv129 mice using bar graphs. Figure 1 and Supplementary Figure 9 were represented using bar graphs. Mice were infected with 2 × 10^6^ *L. amazonensis* Josefa promastigotes in the right hind footpad. The lesion development was monitored vertically **(A)** and horizontally **(B)** using a caliper on the indicated days until the 114th day post-infection. **(C)** The lesion area was evaluated as described in Method. Data are representative of two independent experiments (mean ± standard deviation; *n* = 5).).^***^*P* < 0.001, ^**^*P* < 0.01, ^*^*P* < 0.05; assessed by Student's *T*-test.

Supplementary Figure 11 Lesion development in *Leishmania amazonensis*-infected Sv129 mice with inoculation doses of 2 × 10^5^ promastigotes. Mice were infected with 2 × 10^5^ *L. amazonensis* Josefa promastigotes in the right hind footpad. Images were taken of the lesion development **(A)** and the footpad thickness was monitored using a caliper on the indicated days until the 178th day post-infection **(B)**. Data are representative of two independent experiments (mean ± standard deviation; *n* = 5).
