# Supplementary figures and images for: Characterization of Sv129 Mice as a Susceptible Model to Leishmania amazonensis
Source: Front Med (Lausanne). 2019 May 29;6:100. doi: 10.3389/fmed.2019.00100 (PMC6548835; doi:10.3389/fmed.2019.00100)

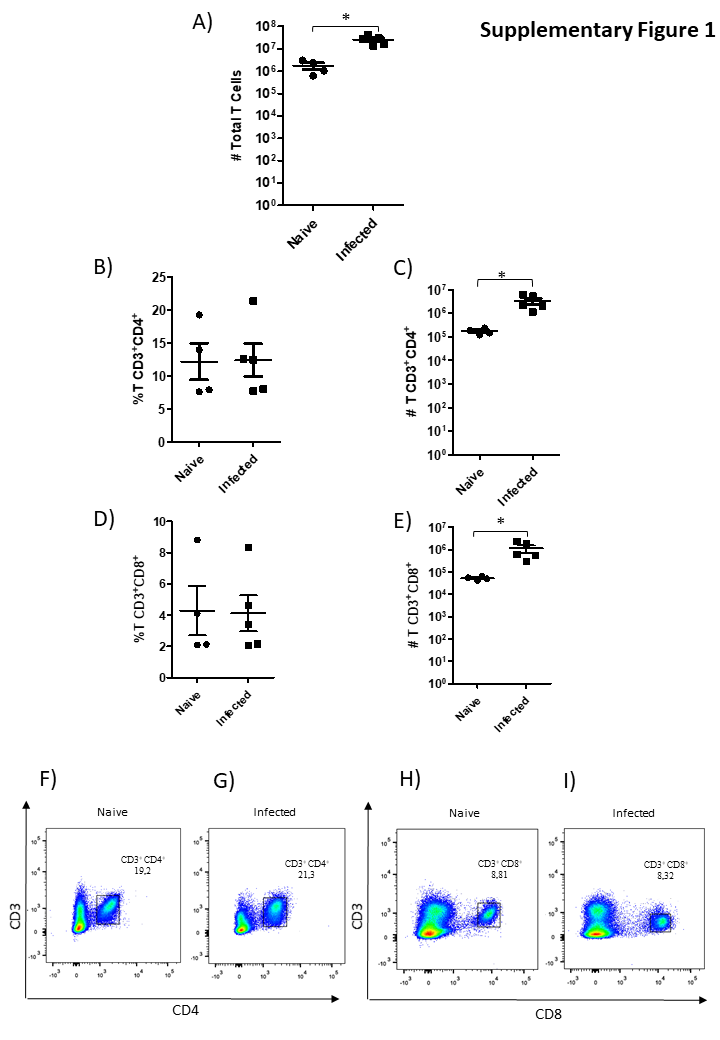

Supplement: Supplementary Figure 1 — Lymph node cellularity, and frequency and number of CD4+ and CD8+ T cell populations from infected and naive Sv129 mice. Mice were infected with 2 × 10^6^ L. amazonensis Josefa promastigotes in the right hind footpad. Lymph node cells of infected mice and naive Sv129 mice were quantified using a Neubauer‘s chamber (A) (mean ± standard deviation; n = 4–5). Lymph node cells from infected mice and naive Sv129 mice were plated at 1 × 10^6^ per well and stained for flow cytometry; frequency and total of CD4+ cells (B,C,F,G) and frequency and total of CD8+ cells were stained (D,E,H,I). Data are representative of four independent experiments (mean ± standard deviation; n = 4–5) *P < 0.05; assessed by Student's T-test. [file Image_1.TIF]

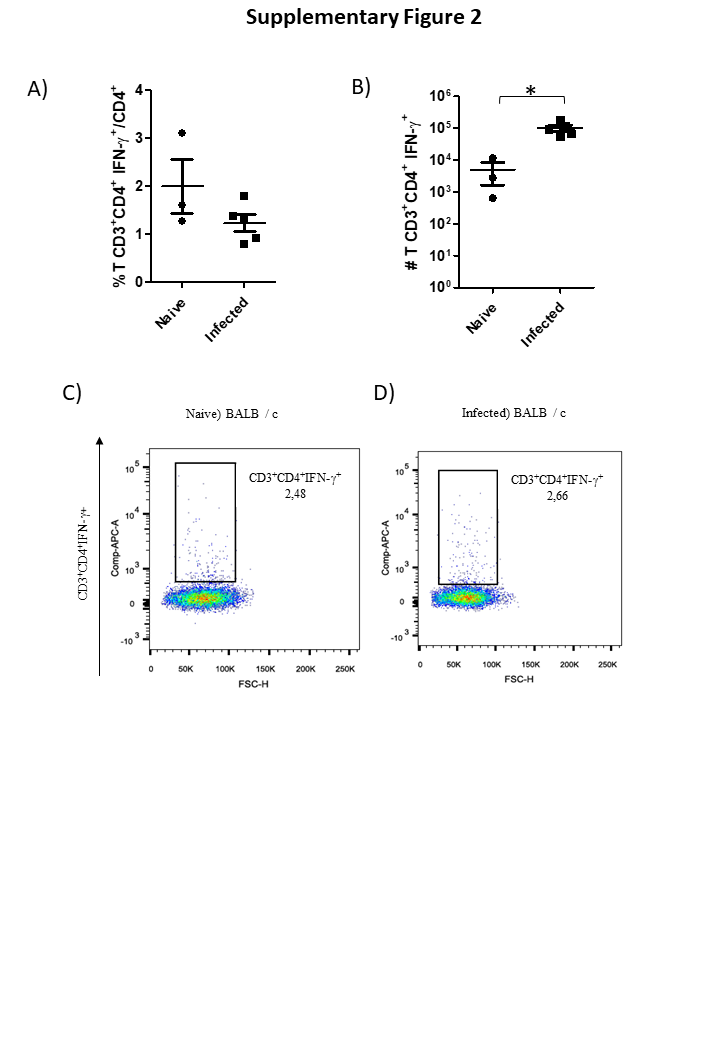

Supplement: Supplementary Figure 2 — Infected BALB/c mice have an increase in the number of IFN-γ-producing CD4+ T cells. Mice were infected with 2 × 10^6^ L. amazonensis Josefa promastigotes in the right hind footpad. Lymph node cells of infected and naive BALB/c mice were plated at 1 × 10^6^ per well and re-stimulated for 4 h with PMA (20 ng/mL) plus ionomycin (1 μg/mL) and then stained for flow cytometry. The percentage of CD4+IFN-γ+ (A) and number of CD4+IFN-γ+ (B) cells. Representative dotplots of the CD4+IFN-γ+ population (C,D) (mean ± standard deviation; n = 4–5). Data are representative of three independent experiments producing the same result profile. *P < 0.05; assessed by Student's T-test. [file Image_2.TIF]

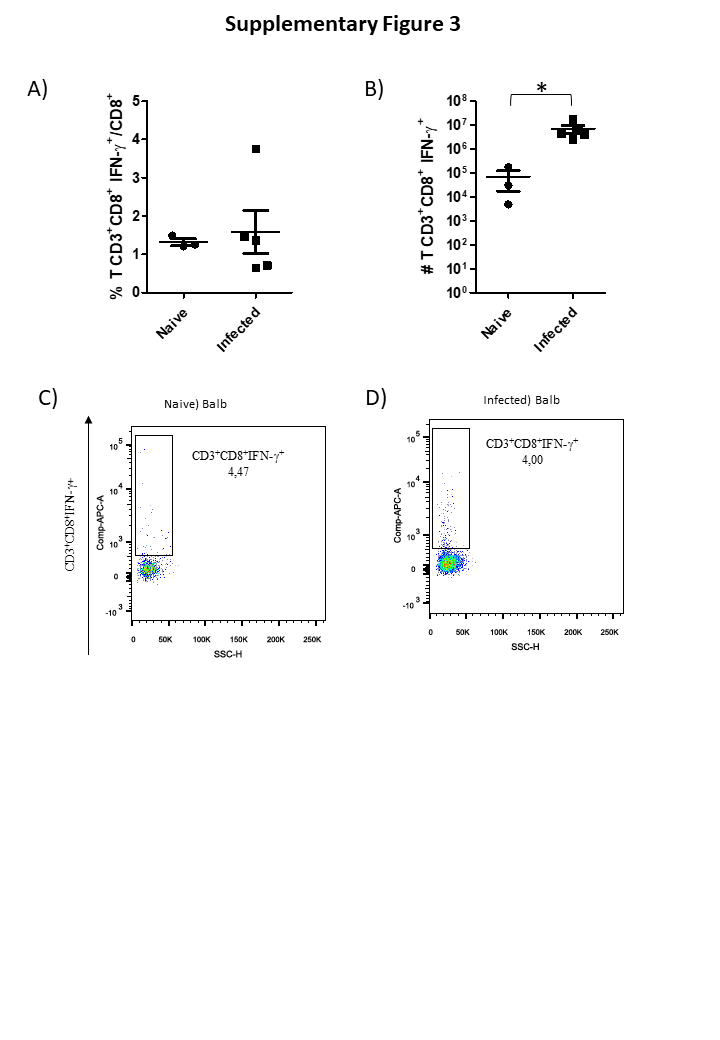

Supplement: Supplementary Figure 3 — Infected BALB/c have an increase in the number of IFN-γ-producing CD8+ T cells. Mice were infected with 2 × 10^6^ L. amazonensis Josefa promastigotes in the right hind footpad. Lymph node cells of infected mice and naive mice were plated at 1 × 10^6^ per well and re-stimulated for 4 h with PMA (20 ng/mL) plus ionomycin (1 μg/mL) and then stained for flow cytometry. The percentage of CD8+IFN-γ+ (A) and number of CD8+IFN-γ+ (B) cells. Representative dotplots of the CD4+IFN-γ+ population (C,D). Data are representative of three independent experiments producing the same result profile (mean ± standard deviation; n = 4–5). *P < 0.05;assessed by Student's T-test. [file Image_3.TIF]

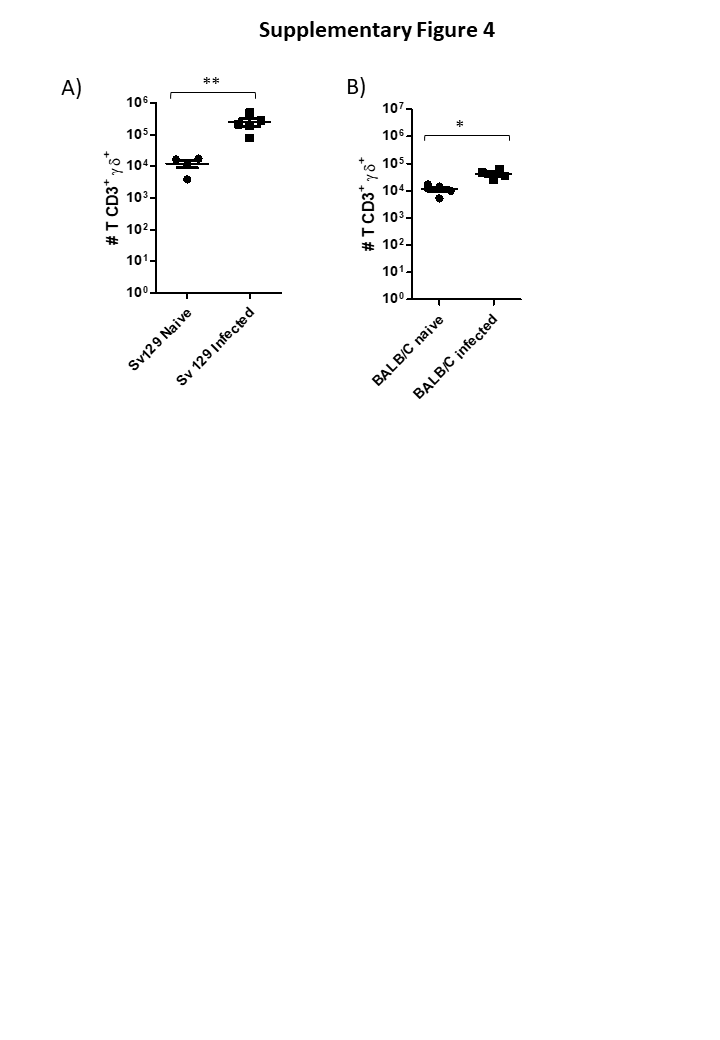

Supplement: Supplementary Figure 4 — Infected Sv129 mice present an increase in the number of γδ T cells. Mice were infected with 2 × 10^6^ L. amazonensis Josefa promastigotes in the right hind footpad. Lymph node cells of infected mice and naive mice were plated at 1 × 10^6^ per well and re-stimulated for 4 h with PMA (20 ng/mL) plus ionomycin (1 μg/mL) and then stained for flow cytometry. The number of γδ T cells in infected and naive Sv129 mice (A) and BALB/c mice (B). Representative of three independent experiments (mean ± standard deviation; n = 6–9). **P < 0.001, *P < 0.05; assessed by Student's T-test. [file Image_4.TIF]

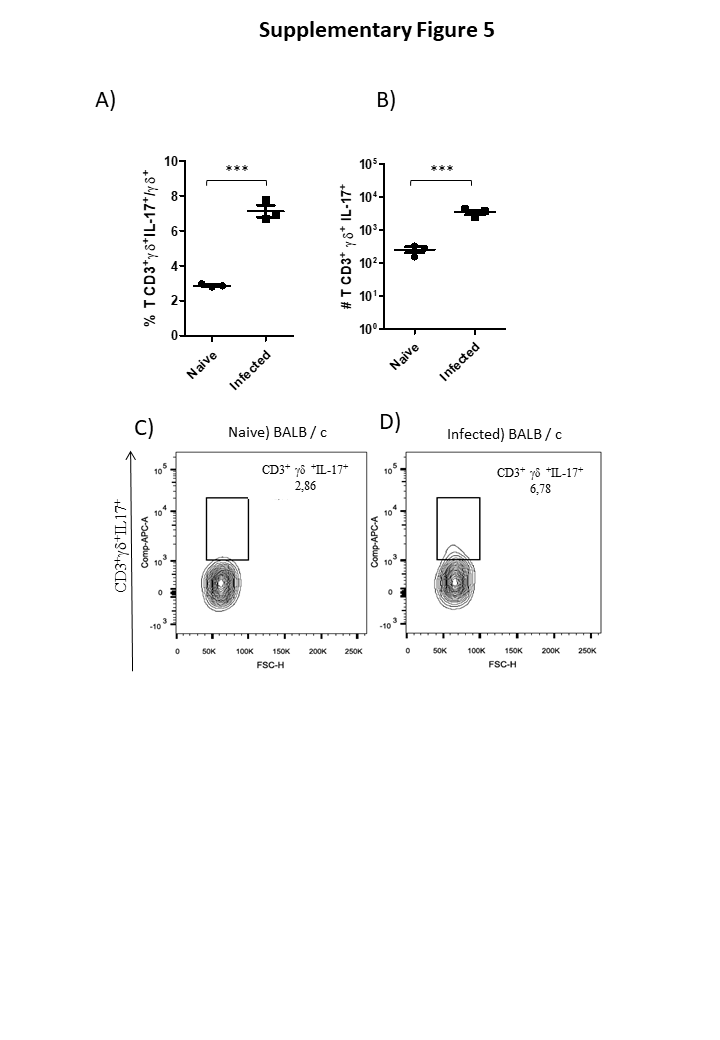

Supplement: Supplementary Figure 5 — Infected BALB/c mice show an increase in the frequency and number of IL-17-producing γδ T cells. Mice were infected with 2 × 10^6^ L. amazonensis Josefa promastigotes in the right hind footpad. Lymph node cells of infected mice and naive mice were plated at 1 × 10^6^ per well and re-stimulated for 4 h with PMA (20 ng/mL) plus ionomycin (1 μg/mL) and then stained for flow cytometry. The percentage (A) and number (B) of IL-17-producing γδ T cells. Representative dotplots (C,D). Data are representative of three independent experiments producing the same result profile (mean ± standard deviation; n = 3). ***P < 0.0002, ***P < 0.0003 assessed by Student's T-test. [file Image_5.TIF]

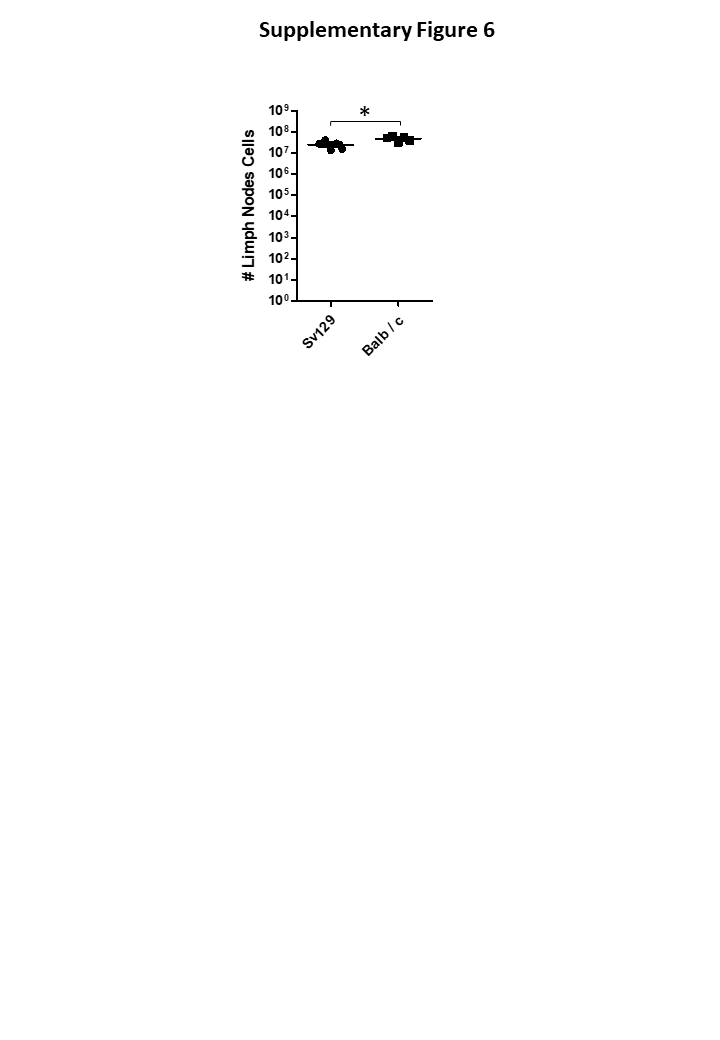

Supplement: Supplementary Figure 6 — Lymph node cellularity in Sv129 and BALB/c mice. Mice were infected with 2 × 10^6^ L. amazonensis Josefa promastigotes in the right hind footpad. Total lymph node cells of infected Sv129 and BALB/c mice were quantified using a Neubauer‘s chamber (A). Data are representative of three independent experiments producing the same result profile (mean ± standard deviation; n = 5). *P < 0.01; assessed by Student's T-test. [file Image_6.TIF]

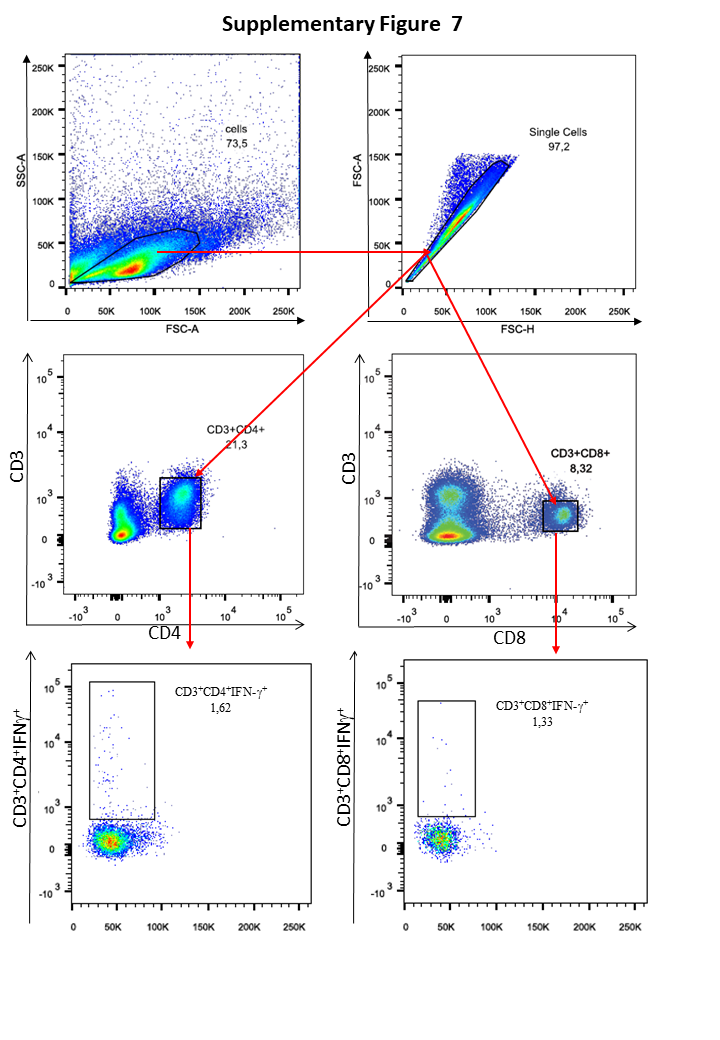

Supplement: Supplementary Figure 7 — Gating strategy used to analyze IFN-γ producing cells. Mice were infected with 2 × 10^6^ L. amazonensis Josefa promastigotes in the right hind footpad. Lymph node cells from infected and naive Sv129 mice were plated at 1 × 10^6^ per well and stained for flow cytometry to determine the percentage of IFN-γ-producing cells. Single cells were gated on CD3+ and CD4+ or CD8+, then on IFN-γ+. [file Image_7.TIF]

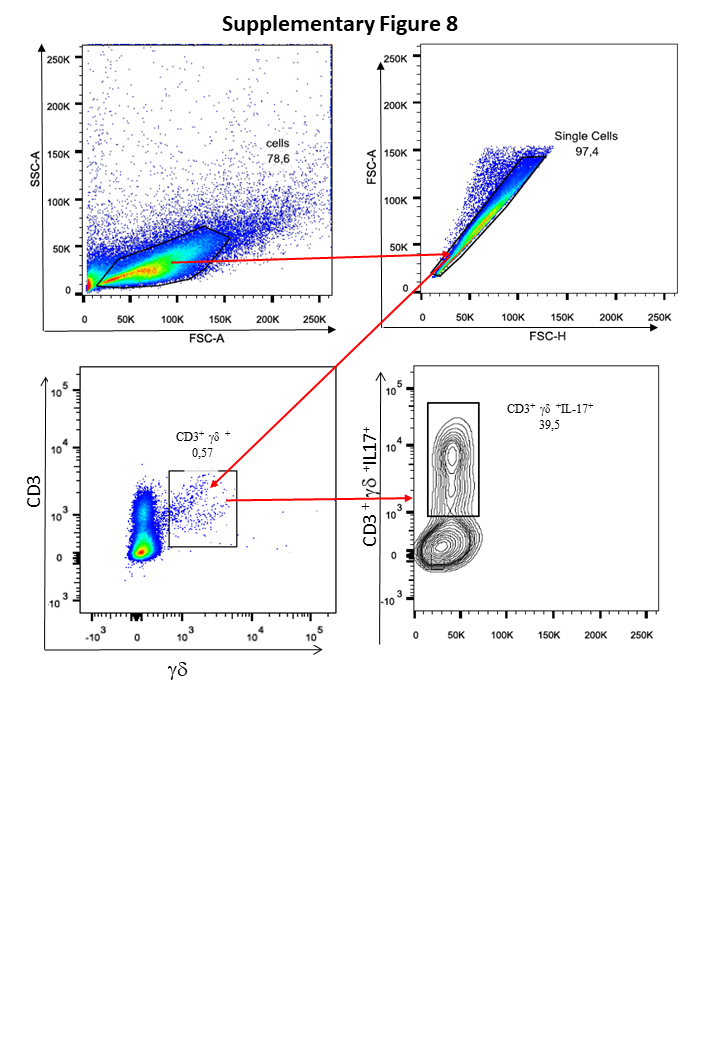

Supplement: Supplementary Figure 8 — Gating strategy used to analyze IL17+ γδ T cells from the lymph node. Mice were infected with 2 × 10^6^ L. amazonensis Josefa promastigotes in the right hind footpad. Lymph node cells from infected and naive Sv129 mice were plated at 1 × 10^6^ per well and stained for flow cytometry to determine the percentage of IL17+ γδ T cells. Single cells were gated on CD3+ and γδ+, then on IL-17+. [file Image_8.TIF]

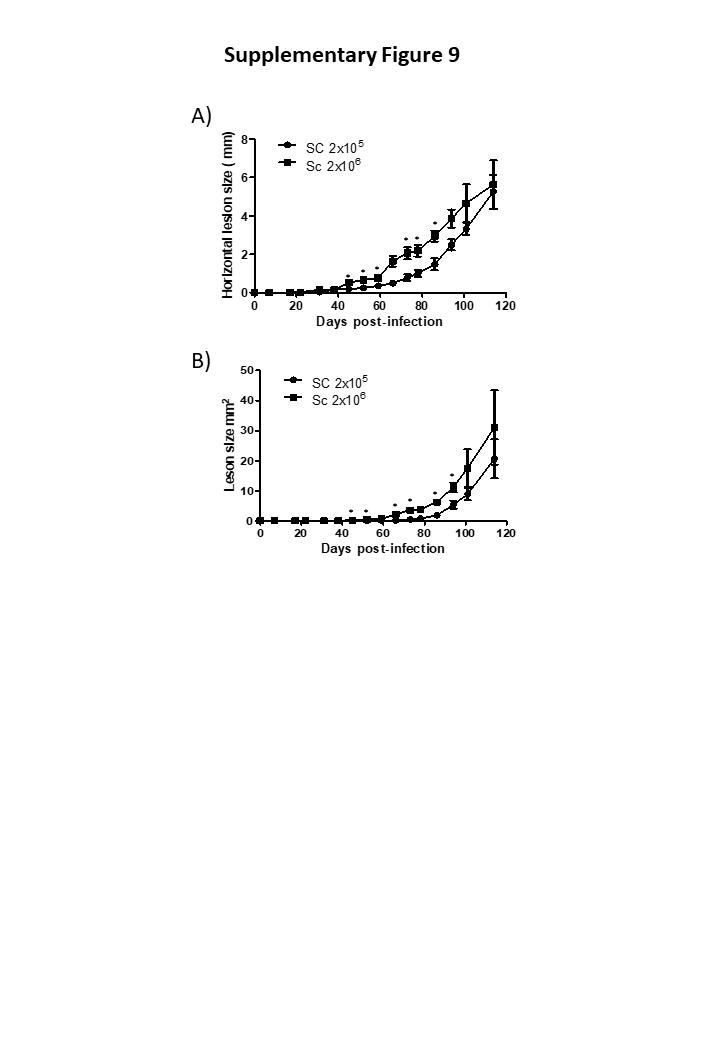

Supplement: Supplementary Figure 9 — Evaluation of the lesion area of Sv129 mice in Leishmania amazonensis infection. Mice were infected with 2 × 10^6^ L. amazonensis Josefa promastigotes in the right hind footpad. (A) The lesion development was monitored horizontally using a caliper on the indicated days until the 114th day post-infection. (B) The lesion area was evaluated as described in Method. Data are representative of two independent experiments (mean ± standard deviation; n = 5). *P < 0.05; assessed by Two-way ANOVA using Bonferroni's post-test. [file Image_9.TIF]

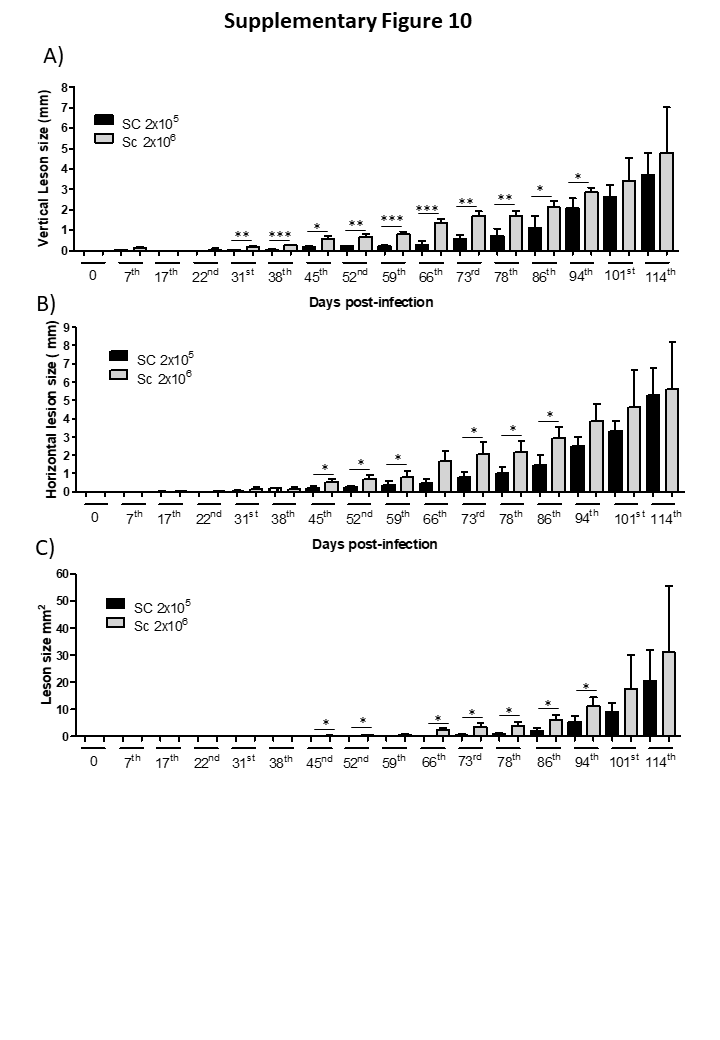

Supplement: Supplementary Figure 10 — Evaluation of the lesion development of Sv129 mice using bar graphs. Figure 1 and Supplementary Figure 9 were represented using bar graphs. Mice were infected with 2 × 10^6^ L. amazonensis Josefa promastigotes in the right hind footpad. The lesion development was monitored vertically (A) and horizontally (B) using a caliper on the indicated days until the 114th day post-infection. (C) The lesion area was evaluated as described in Method. Data are representative of two independent experiments (mean ± standard deviation; n = 5).).***P < 0.001, **P < 0.01, *P < 0.05; assessed by Student's T-test. [file Image_10.TIF]

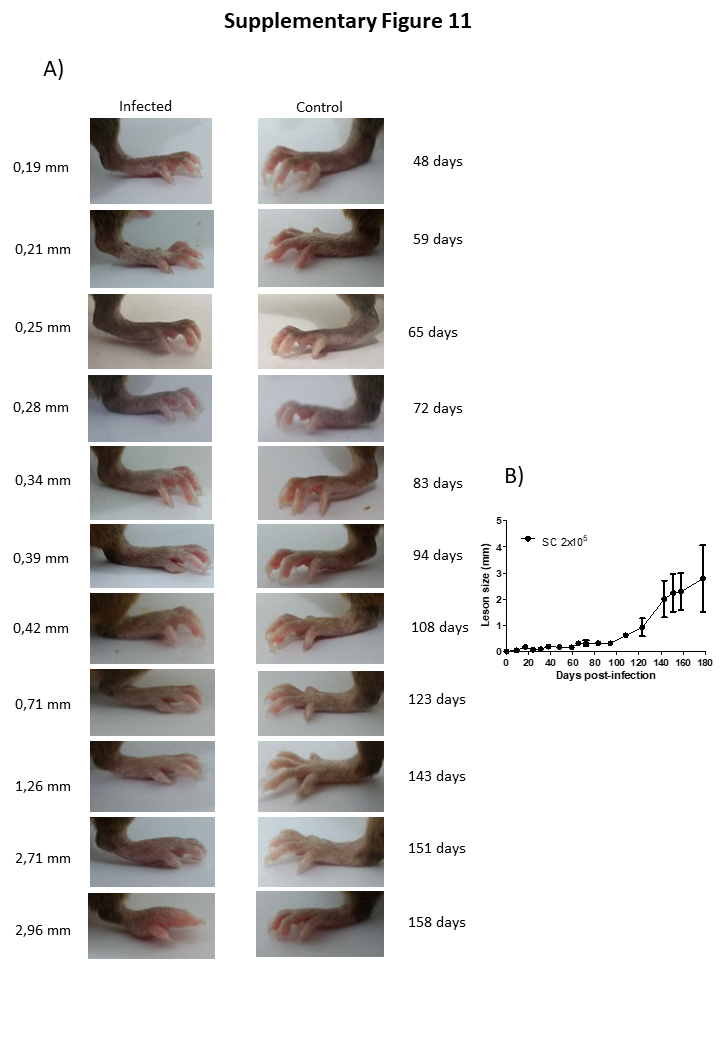

Supplement: Supplementary Figure 11 — Lesion development in Leishmania amazonensis-infected Sv129 mice with inoculation doses of 2 × 10^5^ promastigotes. Mice were infected with 2 × 10^5^ L. amazonensis Josefa promastigotes in the right hind footpad. Images were taken of the lesion development (A) and the footpad thickness was monitored using a caliper on the indicated days until the 178th day post-infection (B). Data are representative of two independent experiments (mean ± standard deviation; n = 5). [file Image_11.TIF]
